# Supplementary material for: Coronavirus Disease 2019 (COVID-19) Pandemic across Africa: Current Status of Vaccinations and Implications for the Future
Source: Vaccines (Basel). 2022 Sep 17;10(9):1553. doi: 10.3390/vaccines10091553 (PMC9504201; doi:10.3390/vaccines10091553)
Supplement: Supplementary file 1 [file vaccines-10-01553-s001.zip › vaccines-1891050-supplementary.pdf]

Supplementary Tables

Table S1. COVID-19 vaccination deployment across African countries: commencement, rollout Programmes, and vaccine sources.

Country	Vaccination Start Date (d/m/y)	Dedicated Rollout Program	Nature of Program			Sources of Vaccines
			Public	Private	Both	
Botswana [232]	26 March 2021	√	√	√	√	• COVAX facility—cost covered by the Government of Botswana<br>• NGOs and COVISHIELD, vaccine donation from the Serum Institute of India<br>• African Union (AU) / African Vaccine Acquisition Trust (AVAT)
Cameroon [233,234]	12 April 2021	√	√			• Donation from the Chinese government<br>• COVAX -WHO<br>• GAVI
Egypt [235,236]	24 January 2021	√	√	√	√	• Donations from different countries including China, UAE, Spain, and Japan<br>• COVAX initiative and in most cases in cooperation with GAVI<br>• Procurements by the government of Egypt facilitated by a loan agreement with the International Monetary Fund (IMF) to support addressing COVID-19′s health and economic consequences<br>• Subsequent plan to produce both China's Sinovac and Sputnik V locally through a joint agreement with both China and Russia
Eswatini [237]	24 March 2021	√	√	√	√	• Oxford/AstraZeneca—India<br>• COVAX<br>• Johnson and Johnson—USA<br>• Pfizer—USA
Ghana [238,239]	01 March 2021	√	√	√	√	• COVAX<br>• GAVI<br>• NGOs<br>• Donations from the governments of Germany, Denmark, Iceland, Norway, and the USA
Kenya [240,241]	08 March 2021	√	√	√	√	• COVAX donations from India, Denmark, France, Greece, Canada, Poland, China, Germany, Argentina, Netherland, Belgium, Slovakia, UK, and USA<br>• Government of Kenya/AVAT<br>• Belgium/AVAT
Malawi [180,242,243]	17 March 2021	√	√	√	√	• Donations by WHO through COVAX facility<br>• Donations from UK government, India, CEPI, GAVI, UNICEF, and ACT-accelerator among others<br>• Donations from African Union Vaccine Acquisition Trust (AVAT)

						• In-country logistic costs covered by the Malawi government with donors help and World Bank
Nigeria [243–251]	05 March 2021	√	√	√	√	• Donation from multi-lateral and corporate organisations—GAVI, COVAX, MTN,<br>• AU, and African CDC<br>• Donations from other countries including USA, France, India, Japan, Italy, and Spain<br>• Procurement by the Nigerian government
South Africa [109]	17 February 2021 *	√	√	√	√	• Bilateral agreement with Pfizer and Johnson and Johnson for local vaccine manufacture<br>• NGO, i.e., Sisonke Programme **<br>• Donations from USA<br>• COVAX-WHO
Sudan [252]	05 April 2021	√	√	---	---	• COVAX-WHO<br>• Donations form UAE, USA
Tanzania [253,254]	26 July 2021	√	√	√	√	• COVAX- WHO and Johnson and Johnson<br>• Donations from China
Uganda [255–257]	10 March 2021	√	√	√	√	• COVAX -WHO<br>• Donations from Danish government, USA, and China
Zambia [258]	14 April 2021	√	√	√	√	• COVAX—WHO<br>• Procurement by the government of Zambia
Zimbabwe [109]	18 February 2021	√	√	√	√	• Donations from the governments of China, Russia, and India<br>• Procurement through the government of Zimbabwe

NB: * Although South Africa was one of the first African countries to receive the COVID-19 vaccine (Oxford/ AstraZeneca COVID-19 vaccine produced by AstraZeneca-SK Bioscience and the Serum Institute of India) on 2021/02/01, the planned roll-out of the vaccine was suspended on 2021/02/08 following evidence that the vaccine had low efficacy against the 501Y.V2 variant [109,259].** Sisonke Programme is a research collaboration between the National Department of Health (NDoH), South African Medical Research Council (SAMRC), Desmond Tutu Health Foundation, CAPRISA, Janssen and Johnson and Johnson [260,261]. Please refer to the principal paper for details of the references.

Table S2. COVID-19 vaccination coverage across African countries (up to the end of 2021).

Country	Age Groups Vaccinated and Order of Priority	Children Vaccinated	Commencement of Booster Doses and Target Groups
Botswana	Adults ≥18 years	≥12 to <18 years planned for Phase IV of the roll-out	*Commenced on 17 January 2022*<br>• Adults ≥18 years previously vaccinated<br>• Pfizer (≥6 months after completing vaccination series)<br>• Moderna (≥6 months after completing vaccination series)<br>• Johnson and Johnson (≥2 months after completing primary vaccination dose)<br>• Oxford/AstraZeneca (≥3 months after completing vaccination series)<br>• Sinopharm (≥5 months after completing vaccination series)
Cameroon	Order of priority:<br>(1) HCWs<br>(2) High-risk groups<br>(3) Adults ≥18 years	Children not yet vaccinated	*Booster doses commenced*<br>HCWs and adults >50 years, 3 months after the primary schedule
Egypt	First phase with order of priority:<br>(1) Priority for health workers/medical staff dealing with COVID-19 patients at quarantine<br>(2) Those dealing with other patients (fever, and chest hospitals)<br>(3) Elderly and those with chronic and immune diseases<br>Second phase:<br>• Adults ≥18 years	Order of priority:<br>(1) 15–18 years<br>(2) 12–15 years with informed consent of parent/legally authorized representatives	*Booster doses commenced*<br>Administered in phases with similar order of priority as for the initial doses
Eswatini [167,262]	Order of priority:<br>(1) Adults ≥60 years with co-morbidities<br>(2) Adults ≥18 years<br>Children ≥12 years	≥12 years	Booster doses commenced late 2021/early 2022 with the Oxford/AstraZeneca vaccine added as a booster to list of vaccines available late January/ early February 2022
Ghana [263,264]	Order of priority:<br>(1) HCWs<br>(2) Adults ≥60 years and those with underlying health conditions<br>(3) Frontline executive, legislature, judiciary, and their related staff<br>(4) Frontline security personnel	≥15 to <18 years	*Commenced January 2022*<br>Target groups:<br>• HCWs<br>• ≥ 60 years<br>• Frontline security personnel<br>Members of the executive, judiciary, and the legislature

	(5) Some religious leaders and other personalities		
Kenya [265,266]	Adults ≥18 years with priority given to essential workers, i.e., HCWs, security officers, teachers, older persons, and those with co-morbidities	≥15 to <18 years	*Booster doses commenced late December 2021*<br>• Fully vaccinated can receive an additional dose of either the Oxford/AstraZeneca, Moderna or Pfizer vaccine 6 months after completion of the primary series<br>• By 22 January 114,007 booster doses had been administered
Malawi	Order of priority:<br>(1) HCWs, police, immigration, Malawi Defence Force, prison warders, prisoners, teachers, adults ≥60 years, and those with underlying conditions considered at the highest risk<br>(2) Adults ≥18 years	*As of November 2021*<br>≥12 years	No booster doses yet
Nigeria [267]	Adults ≥18 years with priority to HCWs and people at risk with co-morbidities	Children not yet vaccinated	*Commenced early December 2021*<br>Eligibility criteria is any adult ≥18 years ≥6 months after receiving the second dose of Pfizer, Oxford/AstraZeneca, or Moderna vaccine
South Africa	*Phase 1: Sisonke 1 Programme targeting 1.2 million HCWs*<br>17/02/2021 to 16/05/2021<br>*Phase 2: National roll-out with order of priority:*<br>(1) 17 May 2021: ≥60 years<br>(2) 01 July 2021: ≥50 years<br>(3) 01 August 2021: 35–49 years<br>(4) 20 August 2021: 18–34 years<br>(5) 30 August 2021: Eligible pregnant and lactating women	≥12 years <18 years<br>1) 20 October 2021: 1st dose of Pfizer vaccine<br>2) 09 December 2021: 2nd dose of Pfizer vaccine	*Commenced 8 November 2021*<br>(1) 8 November 2021 to 1 February 2022: Johnson and Johnson vaccine for all HCWs who were part of Sisonke 1, 6 months after the primary dose<br>(2) 17 February 2022: Immunocompromised individuals eligible for an additional booster dose:<br>• Johnson and Johnson: 60 days after the booster (2nd) dose<br>• Pfizer: 90 days after receiving the booster (3rd) dose<br>(3) 26 February 2022: ≥18 years<br>• Johnson and Johnson: 60 days after primary dose<br>• Pfizer: 180 days after receiving the 2nd dose
Sudan	Order of priority:<br>(1) HCWs and any person with a chronic disease<br>(2) Adults ≥45 years and adult travellers<br>Currently: All adults ≥18 years	Children not yet vaccinated	No booster dose yet
Tanzania	Only adults ≥18 years eligible	Children not yet vaccinated	No booster dose yet

Uganda [268–271]	Started with HCWs, security officers, journalists, the elderly, and teachers. The intention was to double vaccinate key personnel as soon as possible.<br>Strategy changed in March 2021 to increase the number receiving single doses in view of its effectiveness	≥12 to <18 years<br>Order of priority: Those with co-morbidities<br>Will be extended in 2022 to all	*Commenced December 2021*<br>Adults ≥50 years and those with co-morbidities, to further enhance their immunity, 6 months after primary schedule
Zambia [272]	Adults ≥18 years<br>Priority: HCWs	*As of January 2022*<br>≥12 to <18 years	*Commenced 27 December 2021*<br>Available for everyone <18 years
Zimbabwe [109,273,274]	Adults ≥18 years targeted initially	*As of November 2021*<br>≥16 to <18 years	*Commenced end December 2021*<br>Priority are HCWs 6 months after receiving their second vaccine dose

NB: HCWs: healthcare workers; Available published data up to the end of 2021; extended occasionally for more reliable data. Please refer to the principal paper for details of the references.
